# Functional foods as a formulation ingredients in beverages: technological advancements and constraints

**DOI:** 10.1080/21655979.2021.2005992

**Published:** 2021-12-19

**Authors:** Shagun Sharma, Astha Singh, Swati Sharma, Anil Kant, Surajbhan Sevda, Mohammad J. Taherzadeh, Vijay Kumar Garlapati

**Affiliations:** a Department of Biotechnology and Bioinformatics, Jaypee University of Information Technology, Solan, India; b Department of Biotechnology, National Institute of Technology Warangal, Warangal, India; c Swedish Centre for Resource Recovery, University of Borås, Borås, Sweden

**Keywords:** Functional beverages, probiotic strain, nutritional value, immune system

## Abstract

As a consequence of expanded science and technical research, the market perception of consumers has shifted from standard traditional to valuable foods, which are furthermore nutritional as well as healthier in today’s world. This food concept, precisely referred to as functional, focuses on including probiotics, which enhance immune system activity, cognitive response, and overall health. This review primarily focuses on functional foods as functional additives in beverages and other food items that can regulate the human immune system and avert any possibility of contracting the infection. Many safety concerns must be resolved during their administration. Functional foods must have an adequate amount of specific probiotic strain(s) during their use and storage, as good viability is needed for optimum functionality of the probiotic. Thus, when developing novel functional food-based formulations, choosing a strain with strong technological properties is crucial. The present review focused on probiotics as an active ingredient in different beverage formulations and the exerting mechanism of action and fate of probiotics in the human body. Moreover, a comprehensive overview of the regulative and safety issues of probiotics-based foods and beverages formulations.

## Graphical abstract

**Figure:**
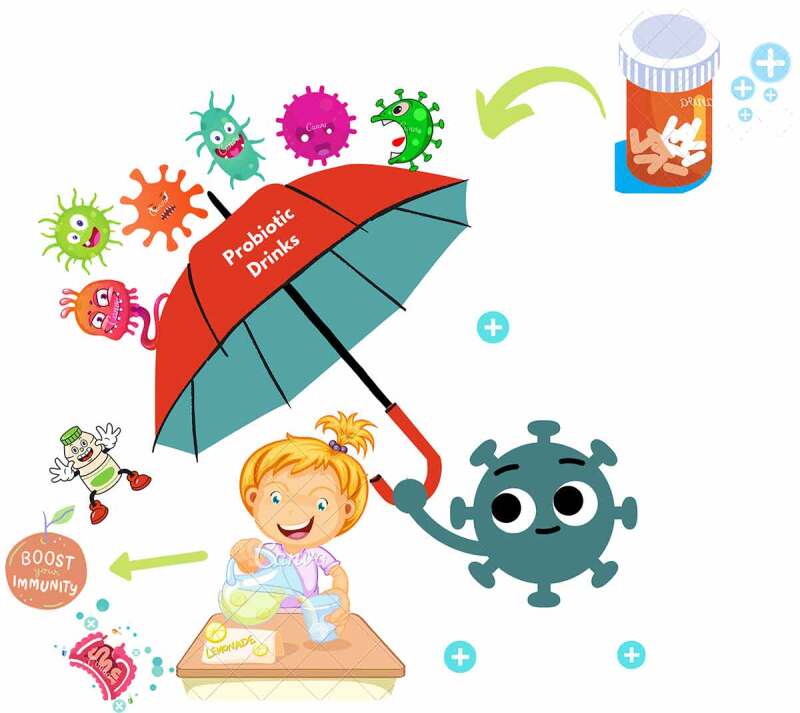

## Highlights

- Nutritional status is one of the leading risk factors for health ailments.
- Probiotics-based functional food is one way of nutritional enrichment of beverages. 
- Probiotics reduce intestinal inflammation by NF-κB activation inhibition.
- The process-based parameters pH and temperature, affects the probiotic formulation.
- Present impinged ”Immunity” awareness boosts the technological sector.   

## 1. Introduction

Due to modernization and a growing movement toward a healthy society, customers are more annoyed with exercise and diet, including food protection, to improve their living standards. The sustainable development goals of UNO (Goal 3) also impinges the well-being of individuals of all ages is a prerequisite to combat the pandemic outbreaks such as COVID-19 (https://www.un.org/sustainabledevelopment/health/). Nutritional status has been described as one of the leading risk factors for severe illness, including corpulence and malnutrition, when the economy is struggling with a global pandemic of sudden acute respiratory syndrome coronavirus 2 (SARS-CoV-2) [1]. Hence in the current scenario, the only rational options for survival are safe, nutritious, and usable foods, which play an essential part in improving the immune system’s ability to fight the disease, thereby improving health [2].

Although conventional preserved foods or fermented foods and drinks have been considered synonymous with certain indigenous practices as per the culture and are recognized as playing an essential part in ancient civilization and diversity that is commonly recognized across countries and continents [3], initially these traditional food items were produced without taking into account the scientific application behind along with the role played by microorganisms. However, presently as the consumer consensus has grown with time, multiple shareholders in food networks are deviating from the traditional outlook of food processing by adding or increasing the nutritious and health advantages that make food items more effective and practical. These nutraceutical food items exceed essential nutrition and provide nutritional benefits beyond the generally balanced diet. Hence they’re also referred to as the ‘future foods.’ Further ahead, for selling these as functional foods, they must fulfill many requirements, including validation and meeting the local food safety regulations or supplying these outsides and adhering to international food quality standards, as well as providing free accessibility and evidence of nutritional benefits when eaten as a part of a regular meal. Presently, probiotics, prebiotics, antioxidants, herbal supplements, dietary fiber, minerals, and other phytochemicals dominate the functional foods sector [4–6].

Probiotics-based food items and drinks are accounted for as one of the potential functional foods that are pretty popular and have a broader acceptance by customers among new functional foods on the market [7]. When consumed in sufficient quantities, probiotics have different effects on the body, improving the host’s health by providing nutritional benefits [8]. This outcome is anticipated to include the balancing of the intestinal homeostasis by suppressing or inhibiting microbes [9,10], strengthening the immunity [11] to minimizing the chance of developing various cancers [12], improving the assimilation of lactose [13] as well as other advantages such as preventing the risk of heart diseases, diabetes, and allergic reactions [14]. Furthermore, after the ingestion of probiotics, there are modifications to the intestinal tract microbiota [15,16]. Probiotic strains have been included in fermented foods and medications in the past as well. Probiotics are the integral parts of different fermented foods (Kimchi -Korea, Natto-Japan, Miso-Japan/Korea, Tempeh -Indonesia, Kombucha -China, Mabundu -Tanzania. Sourdough bread-Europe/USA, Sauerkraut – Europe). Different fermented dairy ingredients, such as cheese, tofu, soured/cultured milk, and drinking yogurt, have been the market’s most well-known probiotic distribution vehicles [17,18]. This can be attributed due to their distinctive physicochemical and nutritive capacities, which enable these to buffer the stomach’s harsh acidic environment (where pH is 2–3), allowing a possible amount of probiotics to survive in the lower gut and potentially exert their curative effects [19,20]. Yakult, composed of probiotic *Lactobacillus casei Shirota*, was the first fermented dairy beverage [21].

While several probiotic strains have been shown to have beneficial effects on human immunity and overall health, any negative consequences should be seen in distinction from the standpoint of public welfare. To determine chiefly the protection of food items and drinks which are probiotics-based, factors such as their source and composition, infectivity, delivering mechanism, capacity to bear antibiotic-resistant genes or not, the extent of subjection to exposure, host health condition, and planned usage must all be taken into account [22]. In several nations, regulatory frameworks have been established to shield consumers against any misleading claims made about probiotics’ intended use, in addition to protection. Depending on the planned use of probiotic ingredients, population trends, and consumer patterns, numerous regulatory assertions govern the commercial market of several nations [23].

## 2. Role of probiotics in human health and necessity toward recombinat strains

It is essential to have a thorough understanding of the probiotic strain utilized in a product. This is a crucial initial step that is taken into account in vitro studies. In light of this, some researchers have been done on probiotic resistance to human microbiota and different circumstances like the pH of the human gut is acidic ranging from 1.5–3.5 with other gastric juices and bile juices secreting inside when the probiotic is in transit and in conclusion from all the studies an ideal probiotic strain is the one that can oppose the gastric acidity, is rigid against the bile acid, is capable of binding to mucus and epithelial cells or the cell lines, can act against microorganisms that can harm the body, can reduce pathogen adherence to body surfaces, viability enhancement and bile salt hydrolase activity [24–27].

As a result, numerous bacterial species with the potential to become probiotics are studied. As per research, some *Lactobacilli* strains have been proven to reduce antibiotic-associated diarrhea [28]. As a probiotic, Lactobacillus species are often chosen because they exhibit several essential characteristics, including a good level of resistance to acid and bile, the efficiency of adhering to intestinal surfaces, enduring low pH level and gastric juice, showing antimicrobial activity, and resistance to antibiotics, and the capacity to produce exopolysaccharides and eliminating cholesterol [27,29]. *Lactobacillus rhamnosus* CRL1505 has been shown to reduce viral pulmonary harm by modulating immune-coagulative responses and eliminating respiratory viruses [30]. *Lactobacilli* strains commonly used as probiotics comprise *L.casei, L. acidophilus, L. rhamnosus, L. paracasei, L. johnsonii, L. delbrueckii* subsp. *bulgaricus, L. plantarum L. fermentum* and *L. brevis* [31].

Apart from different Lactobacilli strains, Bifidobacterium strains are frequently employed as probiotic bacteria because they have a wide range of bile salt tolerance mechanisms, which is significant as the probiotic bacteria’s positive effects must be synthesized even in the vicinity of various biological fluidic surroundings. While bile resistance varies by strain, wild-type Bifidobacterium and Lactobacilli strains are susceptible to bile salts. They may be rendered tolerant by sub-culturing them in modest bile concentrations and steadily rising the concentrations, gradually making them resistant to bile fluids [29]. Bifidobacterium strains commonly used as probiotics are *B. adolescentis, B. adolescentis, B. infantis, B. animalis, B. bifidum, B. breve, B. longum*. Different companies frequently develop scientifically sounding marketing labels as their trademark names for a few of these bifidobacteria.

**Figure 1.**
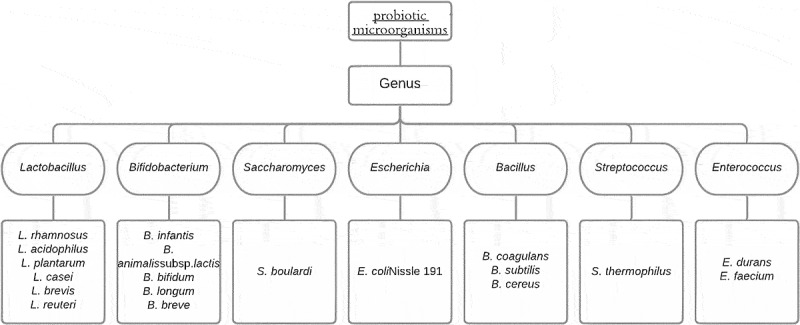
Commonly used probiotic strains

Other than these two prominent strains, a different bacterial genus that can be used as potential probiotics includes (i) Saccharomyces genus, where *S. boulardii* is frequently sold as a lyophilized probiotic for the treatment of diarrhea, and it has a good safety record as well [32]. Other Saccharomyces strains include *S. cerevisiae* utilized in the production of wine, bread, and beer, *S. bayanus* used in the production of wine, and S. boulardii used as a probiotic in medicine. Saccharomyces yeasts also frequently make kefir [33] by forming symbiotic networks with bacteria, and they’re occasionally found in kombucha as well [34]; (ii) Bacillus genus where *B. subtilis* are being explored for animal feeding [35,36] and is recommended for treating diarrhea and eradicating *H. pylori* in people, however according to specific reports there is a risk of consumption of *B. subtilis* spores in o immunocompromised patients, and only under normal host circumstances [37], consumption of these spores is exclusively safe for humans; (iii) *Escherichia* genus where E. coli is prominently known for being a lower intestinal resident and has its probiotic strain referred to as *E. coli* Nissle 1917 (EcN) which in combination with other probiotics, is known to be effective in the treatment of constipation [38] and inflammatory bowel infections [39]. However, further study is needed and may treat gastrointestinal disorders such as Crohn’s disease or ulcerative colitis [40] and colorectal cancer [39]. The commonly used probiotic strains have been summarized in Figure 1.

The Department of Food and agriculture has defined probiotics as live microorganisms, confer a health benefit on the host when consumed in suitable amounts. Concerning health benefits, in adults, probiotics are used as therapeutic agents against gastroenteric microorganisms, lactose intolerance, metabolic disorders, protection against infections (vaginal/urinary tract), repair mechanisms stimulation of cells, ulcerative colitis, production of antimicrobial factors, cholesterol reduction attributes, irritable bowel syndrome, diarrhea associated with antibiotic, colorectal cancer, and control of obesity. In infants, consumption of probiotic aid perinatal health [41,42]. Despite all the scientific advancement, the main question arises: Can a probiotic protect against a specific disease knowing that the mode of action of probiotic action is nonspecific and nondiscriminatory against particular pathogens. In such cases, bioengineered probiotics are used for targeted/specific action pertain to specific disease prevention [43].

Bioengineered probiotics have been in use for over a decade; these are designed for the delivery of therapeutic molecules such as peptides, enzymes, cytokines, DNA, allergens and, single-chain variable fragments [44–46] and application including drug administration, immunomodulation and vaccine delivery [47–54,50]. There are particular attributes required for designing recombinant microorganisms. (1) During product manufacturing and storage should be able to tolerate stress, (2) Under the gastrointestinal environment able to express target antigen, (3) puissant antipathogen, and (4) robust mucosal colonization.

Most of the commonly used probiotics like *Lactobacilli* and *Bifidobacteria* are sensitive to different forms of stress. To confer the problem, stress tolerance can be attained through the persistent sublethal exposure of definite stresses or the genes alteration approaches. The methods are being successfully applied to Lactobacillus and Bifidobacterium strains, with various studies showing the adaptive response to temperature [51], oxygen content [52], and acid stress [53]. The enhanced pathogen-specific activity of probiotics has been reported through the cloning and expression of toxin-specific host cell receptors, i.e., cloning of glycosyltransferase genes (*N. meningitides/C. jejun*i) and expression of chimeric lipopolysaccharide on the engineered probiotic *E. coli for imitation of* host cell receptor of cholera toxin which aids in the treatment of enterotoxin-associated with diarrhea [54].

## 3. Probiotics as a functional ingredient in different beverages

The beverage is a liquid drink intended for human consumption to satisfy human thirst and constitutes human culture. Functional beverages are often referred to as nutraceuticals or as designer beverages [55]. Value-added drinks presently have become quite prevalent in the Westward culture. Today, protein-rich beverages, including sports drinks, dominate the dairy-centered beverage sector [56]. Previously, cheese whey, a by-product of cheese manufacture, mainly was used to make low-value-added items, but after its functional characteristics and high nutritional content were discovered, a wide variety of these have become accessible throughout the global markets, being used to manufacture relatively high-value products. These in particular often tend to comprise various types of drinks, including fermented beverages or non-fermented beverages or beverages prepared with probiotic strains or prebiotics or whey- fruit juices and so on. The dairy-based industry is dominated by nutritional drinks, which are liquids fortified or enhanced with functional additives such as bioactive peptides or minerals and vitamins, prebiotics, probiotics, etc. These utilitarian dairy drinks focused on probiotics, or prebiotics, were the first to be commercialized, and they continue to dominate the sector [57]. Probiotic-based beverages with dairy-based foundations, whey-based foundations, and buttermilk-whey-based foundations, as well as probiotic strains used in the fermentation of some juices, are undoubtedly the most common on the market today.

### 3.1 Dairy-based formulation

Dairy-based probiotic drinks have been accessible in worldwide beverage retails for quite some time now. Fermented milk (drinkable and spoon-able), yogurt, and to a smaller degree, cheese have been used as probiotic carriers in dairy foods for an extended period [58–60]. Probiotic yogurt has become one of the most popular value-added fermented foods available in the last two decades or so. The processing of yogurt necessitates the acidification of milk, which results in the formation of curd. Furthermore, yogurt production is an acidification method that is heavily reliant upon the development of native probiotic LAB (e.g., *Lactobacillus paracasei, Lactobacillus acidophilus, L. delbrueckii, L. casei*, and *Bifidobacterium lactis*). Probiotic yogurt containing potential strains of that of probiotic has been shown in several trials to have several health benefits, including decreasing cholesterol levels in the blood, reducing blood pressure and heart rate, and having an antihypertensive impact [61–63,14].

**Figure 2.**
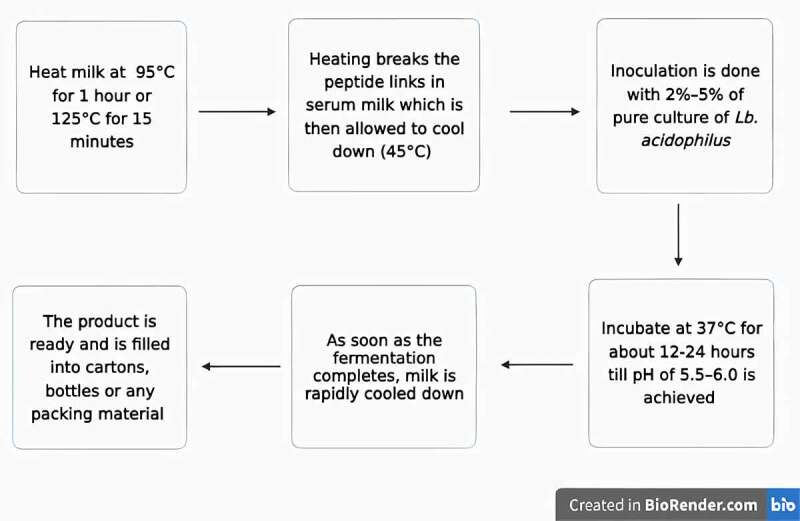
Steps involved in making of acidophilus milk

Acidophilus milk is made from milk that has been heated to a high temperature for an extended period (95°C for 1 h or 125°C for 15 minutes). Extreme thermal processing destroys the proteins and peptide linkages in serum milk, which are critical for the development of *Lb. acidophilus*. Following the thermal processing, all spores’ temperature is lowered to 37°C and left there for 3 to 4 hours to enable all spores to germinate. After that, the milk is reheated again; it further suppresses the actively growing and reproducing microorganisms. Fermentation occurs at 37°C for 18–20 hours, or before the pH reaches 5.5 or 1% lactic acid [64] (Figure 2). The average *Lb. Acidophilus* inoculation rate is 2–5% but replacing 25% of the Lb. Acidophilus with a yogurt culture mixture is recommended in certain situations. It has been proposed that probiotic fermented milk reduces plasma cholesterol levels, lowers low-density lipoprotein (LDL) levels in hypercholesterolemic people, blood pressure, plus hypertension prevention [65]. Probiotic milk has also been shown to help hypertensive and prehypertensive people lower their blood pressure [66].

### 3.2 Whey-based formulation

There has been a surge in using waste materials to create functional drinks in the last few years, with whey being the most notable example. Currently, whey is considered a cheese production by-product but rather a simple ingredient for making enhanced products with much greater value, like probiotic drinks. It comprises a variety of nutritious compounds, including dissolvable milk proteins, minerals, and lactose, and provides an ideal nutrient matrix for probiotic strain’s growth and viability [67–69]. Whey beverages with probiotics being their basis and formulations for the enriched whey-based beverages have governed whey-based beverage studies over the last decade, as it has been exhibited that supplementing these with probiotics or, in fact with the prebiotics has certain health advantages, like reducing hypertension [70]. There are multiple ways of using whey to make healthy probiotic drinks. It can be utilized directly, combined in addition to powders with a dairy foundation, or applied to beverage formulations in various ratios. Several experiments have looked at using whey during LAB fermentation to make lactic probiotic drinks, and probiotic bacteria have also illustrated that they can survive in whey [34]. Numerous prebiotic additives can be operated in the food formulations to facilitate the advancement of the probiotic bacteria in whey-based drinks. The variety and the amount of prebiotics must be deliberately chosen not to harm the sensory properties of the finished product.

During the research, kefir grains were used to make a fermented beverage that resembled kefir by replacing milk with whey [71]. Since multiple Lactobacillus yeasts and bacteria were found, the new beverage could be classified as a probiotic. This research opens up new possibilities for using whey with kefir grains. Apart from this, the growing success of fruit-flavored drinkable yogurt worldwide also presents an excellent opportunity to incorporate whey in producing refined fruit-flavored products [72].

### 3.3 Buttermilk whey-based formulation

Probiotic drinks based on buttermilk-whey aren’t much popular compared to the cheese whey drinks or the ones found on milk. It is most likely attributed to the idea that the dairy industries do not discard large quantities of buttermilk or the buttermilk whey relative to the cheese whey. After 28 days of storage [73], developed a probiotic drink made of buttermilk that has been supplemented with different flavors and sources of carbohydrate (like sucrose or sucralose) that had adequate sensorial content along with a large probiotic tally. Due to probiotic strains’ metabolic action on diacetyl, the buttermilk-based probiotic drink had a decreasing amount of diacetyl than the control beverage, which is a distinguishing feature of the drink. It was also stated by [73], that the development and sustainability of *B. animalis* subsp. lactis. was encouraged by flavored probiotic buttermilk beverages.

### 3.4 Probiotics in juices

**Figure 3.**
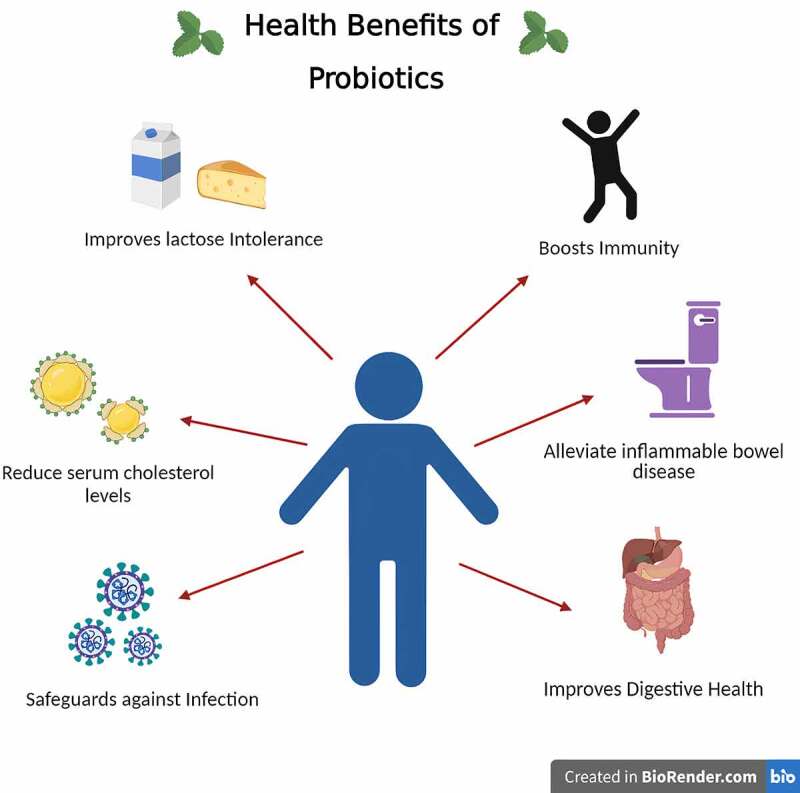
Proposed health benefits of probiotic beverages

Over the years, probiotics have been used in fermented dairy products, but recent studies of alternate raw materials show suitable substrates for nondairy probiotics. Fruit juices are suitable substrates for probiotics because they already comprise valuable nutrients like vitamins, minerals, antioxidants, and fibers. Their natural sugar promotes the growth of probiotics. The consumption of dairy products is also limited as most individuals are lactose intolerant, hypercholesterolemia, or vegan, which eventually leads to more studies on probiotic fruit juices [74]. Another advantage is that the digestion of juices is better than dairy products; they do not have allergens and are naturally cholesterol-free. The most common fruit juices used as fruit matrices for probiotic delivery are cashew apple, pineapple, apple, banana, orange, and blueberry [75–77]. Overall probiotic juices increase the number of probiotic products in the population. The health benefits of probiotics have been depicted in Figure 3.

## 4. Mechanisms of action of probiotics leading to health benefits

### 4.1 Epithelial barrier strengthening

Epithelial cells lining the intestine are constantly in touch with luminal contents and the continually changing enteric flora. Acting as a critical defensive mechanism, the intestinal barrier protects organisms from their surroundings while maintaining epithelial integrity. Increased gene expression involved in tight junction signaling has been a potential strategy for improving the integrity of the intestinal barrier [78–80]. According to recent research, probiotics may restore the barrier role in the body after injury. For example, E. coli Nissle 1917 (EcN1917), apart from inhibiting enteropathogenic *E. coli* from disrupting the mucosal barrier, also restores mucosal integrity in T84 and Caco-2 cells. Therefore, increased production and relocation of the zonula occludens (ZO-2) and PKC tight junction proteins lead to rebuilding the close junction complexes [81,82]. Multiple intestinal disorders have been linked to increased pro-inflammatory cytokines and intestinal absorption [83]. Avoiding cytokine-induced epithelium degradation, which is common in irritant bowel illness, may help strengthen the mucosal barrier when probiotics are used [84]. Mucin glycoproteins (mucins) are significant macromolecular components of epithelial mucus, and they’ve been linked to both wellness and sickness for a long time. Thus, probiotics can increase mucus production to improve the barrier role and prevent infections from entering the system. Human intestine cell lines secrete more mucin when they are in contact with various Lactobacillus species. Although Lactobacillus adherence to the cell monolayer is required for this beneficial role, probably, this would not happen in vivo [85,86]. A minimal number of in vivo studies have been conducted. Therefore there is a lack of consistency at the moment. Probiotics may enhance mucus production in vivo, but further research is required in this domain.

### 4.2 Enhanced cell adhesion

To use a microbial strain as probiotic, several requirements ought to be satisfied. Adherence to the intestinal mucosa enabling colonization and subsequent interaction among the given probiotic strains and the host is a prerequisite [87–89]. Antagonism toward infections and immune system activities are modulated by this unique relationship [90,91]. Thus, adherence has been identified as a critical selection criterion for novel probiotic strains and linked to specific probiotic benefits. Lactic acid bacteria have several surface characteristics that let them associate with intestinal epithelial cells (IECs) and mucus. IECs produce mucin, a complex glycoprotein combination that is the primary aspect of mucous, thereby preventing harmful agents from adhering to it [92–94]. In addition, the mucous gel contains lipids, free proteins, immunoglobulins, and salts. This unique interconnection suggests a probable link amongst probiotic bacteria surface proteins with pathogen inhibition from mucus. Probiotics modify intestinal mucins in a way that prevents pathogens from adhering to them [88,95]. Additionally, the strains of probiotics may also trigger the emission of defensins from epithelial cells, preventing pathogenic infection. These tiny peptides/proteins have antibacterial, antifungal, and antiviral activity.

Furthermore, such tiny peptides/proteins help maintain the intestinal barrier’s overall functioning [96]. Invertebrates, defensins are a large group of membrane-breaking peptides. The contact is nonspecific and occurs mainly via electrostatic interactions with anionic phospholipid molecules on the surface of the membrane. Throughout the bacterial membrane, this contact causes defensin apertures, which undermine membrane strength and lead to the death of microorganisms. Electrostatic interactions allow cathelicidins to attach to membranes of bacteria and, like defensin, cause membrane rupture [97].

### 4.3 Pathogen competitive exclusion mechanism

Competitive exclusion occurs when one bacterium type contends fiercely for receptor sites within the digestive system than most other bacterial species [87,98]. The precise routes and critical support frameworks that underpin probiotics’ actions are largely unclear. Reduced luminal pH, conflict for nutritional resources, and the generation of bacteriocin or bacteriocin-like compounds are some of the primary processes hypothesized for pathogen inhibition [99]. Human pathogens like *Salmonella typhi* and *E.coli* [100] have been the subject of the majority of the research done to date. The regulation of various signaling and metabolic pathways in cells appears to be mediated by certain probiotic compounds. Probiotic metabolome constituents were known to relate with numerous targets in biochemical activities that control the proliferation of the cells, differentiation, programmed cell death, blood vessel formation, and inflammation [101]. Bacteriocins are antimicrobial protein compounds produced by lactobacilli and bifidobacteria that hinder the spread of some diseases.

Further ahead, using probiotics as a preventative or therapeutic measure against enteric infections is referred to as colonization resistance. Each of the 30–60 amino acids in Bacteriocins makes it a little cationic molecule. These chemicals impair the proton motive force by acting on the bacterial plasma membranes and activated membrane vesicles [102].

### 4.4 Modifications of immune system

The gut’s microbiota influences the immune response by producing chemicals with immunomodulatory and anti-inflammatory properties and stimulating immune cells. These effects on the immune system are attributed to probiotic bacteria interacting with epithelial cells, dendritic cells, monocytes/macrophages, and lymphocytes [103]. Probiotics regulate the host immune system as one of their primary modes of action. As a result, the body’s defense system is split into two types: innate and adaptive. B and T cells, which attach to particular antigens, are essential for the adaptive immune response.

On the other hand, the innate system reacts to pathogen-associated molecular patterns (PAMPs), supported by most pathogenic microbes. Pattern recognition receptors (PRRs) that attach to PAMPs are responsible for the primary immune response to infections. Emerging from this, the PRR family also includes the Toll-like receptors (TLR) family, a group of transmembrane proteins found on variousimmune and nonimmune cells, including B- cells, natural killer dendritic cells (NK) as well as macrophages and fibroblasts. In addition, PRRs contain nucleotide-associated oligomerization domains, adhesion molecules, and lectins, among other things [104]. Besides TLRs, PRRs have NOD-like intracellular receptors (NLRs), which defend the cytoplasmic region [105].

Probiotics have been shown to reduce intestinal inflammation by downregulating TLR expression, secreting compounds that prevent TNF from reaching blood mononuclear cells, thereby inhibiting NF-κBactivation in enterocytes. In this way, lactobacilli cell wall constituents may stimulate cytokine production by signaling via TLR2 and TLR6.TLR9 is yet another class of TLRs triggered by probiotics, and it has anti-inflammatory properties at the epithelial lining in vivo. Due to the intracellular signaling pathways caused by TLR9 activation, TNF – stimulated NF-κB is expressed. As a result, various probiotic species are expected to have varied capacities to activate TLR9. NLRs are another type of membrane-bound receptor. While there hasn’t been much research into how probiotics affect these receptors [106], found that L. Salivarius’s protective ability is linked to local IL-10 synthesis, which has an anti-inflammatory effect that NOD2 mediates. Another significant aspect of NLRs is that they initiate a pathway in which NLRs create inflammasomes, activating the adaptor protein caspase 1 required for breaking pro-IL-1 β and pro-IL-18 into physiologically active forms.

**Figure 4.**
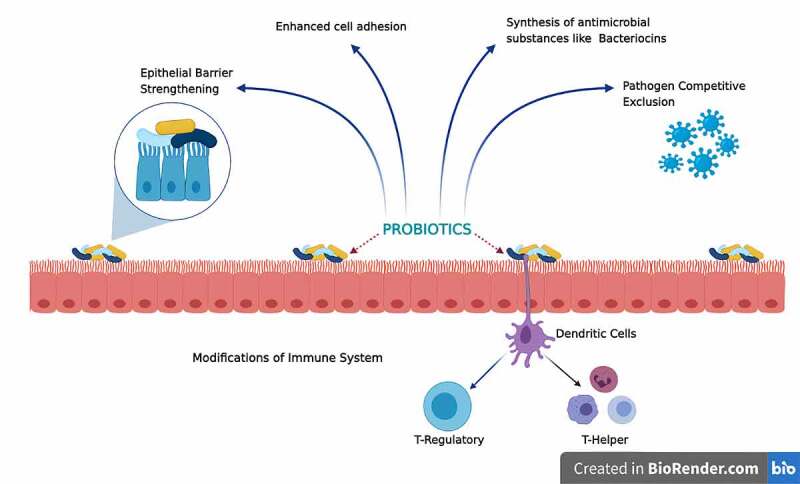
Key mechanisms of action of probiotics leading to various health benefits [Modified and adapted from [87]]

TLR and NOD-like receptors, as well as NF-κB signaling pathways, have recently been characterized in terms of their capacity to promote or inhibit activation and affect downstream pathways. These studies will further throw insight into the intricate interplay of host-microbe interactions. By stimulating these receptors, communal bacteria can induce calibrated antimicrobial reactions that cause slight inflammation and tissue damage in the body. The fundamental mechanisms of probiotics toward different health benefits have been shown in Figure 4.

## 5. ADME (Absorption, distribution, metabolism, and excretion) of probiotics in the human body

**Figure 5.**
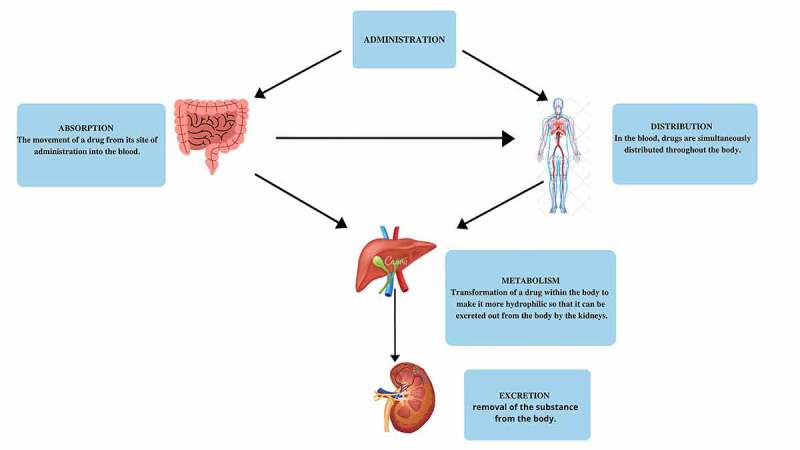
Probiotic ADME (Absorption, distribution, metabolism and excretion) process in the body. [Modified and adapted from [108]]

The misuse of antibiotics resulting in multi-drug resistance microbes has again focused clinical attention on probiotics. Probiotics are living microbes that are regulated as food and drugs. However, the study and testing of probiotics as drugs are still developing, regulated by the Food and Drug Administration. Heedless of the way a probiotic is currently being marketed, it becomes a ‘drug’ for the prevention or treatment of an illness; therefore, probiotics are classified as ‘live biotherapeutics’ under the FDA work definition. For biological probiotics and new pharmaceutical entities, development pathways and requirements are similar, including preclinical tolerability studies, pharmacokinetics, and large, well-controlled clinical trials. However, pharmacokinetic studies such as absorption, distribution, metabolism, and excretion (ADME) are not convenient for Probiotics [90]. When probiotics are taken into the body by oral or intravenous injection, the nutrients carried by probiotics are absorbed from the small intestine (oral administration) or absorbed into the bloodstream (other administration methods). The primary metabolism occurs in the liver and is excreted as urine from the kidneys or feces with bile. Then they pervade blood vessels from the bloodstream and exercise their medicinal and few toxic effects in several organs [107]. For the particular pharmaceutical probiotic major challenge arises to explain factors that may affect absorption, distribution, metabolism, and excretion (ADME) [108]. The ADME process of probiotics has been depicted in Figure 5.

## 6. Health benefit and safety claims of Probiotic beverages

It is said that when a human takes beneficial bacteria via food, it regulates the gut flora resulting in improved health conditions of a consumer. However, a considerable study is required to conclude the same health benefits with an increase in the number of beneficial bacteria [109]. More than 20,000 articles related to the health benefit of probiotics have been published in PUBMED, out of which 2200 were of a clinical trial. The most studied bacteria with over 9000 articles published are Lactobacillus sp., followed by Bifidobacterium sp., Streptococcus sp., Bacillus sp. and Saccharomyces sp. The health impacts of dairy-based probiotic beverages are extensively studied through *in-vitro* cell culture and *in-vivo* animal models, yet clinical trials on humans are still limited.

The maximum number of studies focuses on the effect on the immune system, microbial resistance. The hypocholesterolaemic effect of probiotic strains is studied by *in-vitro* methods, resulting in probiotics that can remove cholesterol by attaching the cholesterol to their cell surface [110]. Miremadi, Sherkat, and Stojanowska had well-reviewed the Anti-hypertensive properties and hypocholesterolaemic effect of prebiotics and probiotics in 2016 in one of their studies found that the daily consumption of probiotics through dairy products will decrease the chances of cardiovascular disease. The viability of *Propionibacterium freudenreichii* 138 and *Lactobacillus casei* BL23 is stimulated under acid, bile salts, and cold storage stress conditions by the augmentation of probiotic skim milk with whey protein isolate at a level of 30% [111]. However, it is found that whey protein isolate increases the anti-inflammatory effect of *Lactobacillus casei* BL23.

*In-vitro and In-vivo* (animal trials) studies do not impart adequate data to conclude the effect of probiotics on the human body as they are only helpful for pilot assessment. The health claims related to humans are only applicable if the proper approach executes them. The joint WHO/FAO union in Cordoba, Argentina, from 1st – 4th of October, 2001, identified the rising demand for probiotic-based products. For the safety of a consumer, it is necessary that before reaching them, the product has gone through the proper assessment and approach. The working groups have made proper guidelines and identified the minimum requirements required to be characterized as ‘probiotic food’ [112]. The commercially used different probiotics have been tabulated under Table 1.

**Table 1.** **Some probiotics products used commercially** [143]

Product	Probiotic strains	Health claim
Biola	*Lb. Acidophilus+ Lb. Rhamnosus+ Bifidobacterium spp.*	Stimulates brains, immune system and nervous system
Enfant Plus (strain Bb-12)	*B. animalis subsp. lactis*	Improve bone health and digestive system
Namyang Jayeonuiflavored probiotic yogurt drink	*Lb. acidophilus*	Help to improve digestive system
Toni Nutri Mix (with Muesli)	*Lb. rhamnosus GG*	Improves immune system and helps slimming
Caldus Milk	*B. bifidum* or *B. longum*	Food for specified health use (FOSHU)
Dreaming probiotic yogurt drink	*Lb. acidophilus + Bifidobacterium spp.*	Stimulate immune system and improve digestive system
Mengniu	*Bifidobacterium spp.*	Stimulates immune system and improve digestive system
Vivita drinking yogurt	*Bifidobacterium spp.+ Lb. acidophilus + Lb. casei*	Help slimming
Yakult	*Lactobacillus casei [strain shirota)*	It helps improve intestinal function also builds immunity
GoodBelly	*Lactobacillus plantarum 299 V*	Healthy digestion
DE111	*Bacillus Subtilis*	Improve immune and digestive system
Lifeway plain kefir drink	*Lactobacillus+ saccharomyces+ Bifidobacterium*	Improve bone health
Mr. Probiotic	*Lb. rhamnosus GG*	Improve digestive system

### 6.1 Microorganisms screening

Isolation and identification are considered to be the initial step for any probiotic used in food implementation. 16s RNA method is used to identify the species and verify with the help of genotypic and phenotypic tests. The presence of Plasmid (extra chromosomal material) supports the strain properties [112,113].

### 6.2 Probiotic potential screening: in vitro

The efficacy of the identified microbe needs to be supported by the in vitro and in vivo tests followed by human clinical trials. There are different categories such as: gastric juice resistance, ability to reduce pathogens in the gut, antimicrobial activity, adherence to human epithelial cells and safety assessment to check whether the identified microbe has a probiotic [113]. To certify the probiotics in beverages, guidelines of FAO/WHO working groups are followed. For the safety assessment following points need to be considered:

1. Production of toxins, undesirable metabolites.
2. Potential side effects in human clinical studies.
3. Chances of antimicrobial activity.
4. Epidemiological studies of the effect on consumers and its assessment.

*In-vitro* studies are more likely to provide more appropriate data on genomic analysis, strain characteristics, viability computation, and DNA- based identification [114].

For the safety assessment, all the approaches should be included in an integrated manner.

### 6.3 *Studying human and animal*: in- vivo

After successful *in-vitro* studies, a sustainable in vivo study is required for the safety interest. Before human trials, approved animal models are used to support in vitro studies. The process for Human clinical studies regarding applying probiotic food is done in four stages: safety evaluation, efficacy, effectiveness, and Surveillance [115].

#### Safety assessment

Detailed screening of isolates is done, and factors like antibiotic resistance pattern, possible undesirable side effects, and measure of toxin production are recognized. All the procedures are followed, which are recommended by the working officials.

#### Efficacy

To evaluate the effectiveness, animal studies are necessary [114]. The animal study results are required to be assessed conscientiously and expounded like morphology, physiology, anatomy, pathology, phytology, test groups, and other factors.

#### Effectiveness

The effectiveness of the probiotic in food needs to be evaluated, and human trials do it. The number of viable cells of the tested probiotic strain in terms of ml/CFU in the carrier drink/ food should be indicated, providing health claims.

#### Surveillance

As safety is a priority, before human trial, the authorizing body should approve it. The study depends on the category of probiotic strain utilized in the beverages and studied population size.

The in-vivo studies of probiotics-based beverages have been summarized under Table 2.

**Table 2.** In-vivo studies of probiotic based beverages

Probiotic based beverage	Animal studies output	Human studies output	References
Kombucha	Mice: Treatment of gastric ulceration; weight loss; decrease in the ratio of retroperitoneal adipose tissue to body weight; decrease in lipid deposition within the liver cells cytoplasmDuck: Decrease in total cholesterol and the LDL-Ch levels; escalating the HDL-Ch levels	Cytotoxic effects observed on the cancer cells; metalloproteinase matrix activity is decreased, as is the cancer cell motility; infiltration of the cancer cells into the host cells is reduced	144,145,146,147,148
Yakult	Male wistar rat: Decreases in sympathetic nerve outflow of anesthetized rats in a tissue-specific manner.	When taken 3 times a week the risk of developing hypertension in eldery people is lowered; probiotic administration altered the gut microbiota and reduced bacterial translocation in Japanese patients with Type 2 Diabetes Mellitus	149,150
Kefir	Broiler chickens: Enhances growth performance; lowers total lipid and cholesterol; reduces serum LDL and increase HDL cholesterol; increases antibody titer against sheep red blood cellsWistar rats: Reduced oxidative stress and hyperglycemia progression	Inhibit angiotensin-converting enzyme activity; controls inflammatory response; enhances digestion and microbiota of gut; decrease level of fasting blood glucose and HbA1C levels therefore can be used as adjuvant therapy in prevention of diabetes	151–155
Probiotic Bitter Melon Juice	Sprague-Dawley rats: Decrease in blood glucose levels, significant increase in superoxide dismutase(SOD]	Known to manage blood sugar levels, reduce fructosamine levels in type 2 diabetic patients [as per some studies)	156
Whey Drinks	Male Sprague–Dawley rats: Decrease of of triacylglycerols and low density cholesterol; increase of high density cholesterol	Vitalize immune system; lowers serum cholesterol level and blood pressure, lowers the threat of cancer	157,158
Goodbelly	Rabbit: Reduce the chances of obesity and metabolic syndrome in rabbit [ITELV2006 rabbit strain]	During antibiotic therapy decreases gastrointestinal symptoms; decrease in inflammatory response	56
Gefilus juice	Mouse: Prevent bone loss and gingival inflammation in the mouse.	Has antitumor activity and activity against toxins	159–161
Mengniu	Mice: Change in behavior and sociability of mice	Stimulates the immune system and improves the digestive system	143,162
Bio-K+ culturedmilk basedprobiotic	Rat: A preventive effect against colon carcinogenesis by decreasing the total number of ACF in DMH treated rats is observed.	Has shown potential antitumor activity	163,164
DanActive	Mice: improves intestinal microbiota thus enhancing gut immune response; increased secretory-IgA in intestinal fluids of offspring mice	Known to reduce overall incidence of common illness like flu, sinusitis, diarrhea and ear infections; shows activity against pathogens bacteria and viruses	145,165

#### Labeling requirements

- Following standard international nomenclature, Genus, species and strain should be labeled.
- Minimum viable number of probiotics and the level at which efficacy is claimed
- Clearly stated health claims
- Serving size for efficacy
- Storage conditions should be mentioned

Probiotics beverages are inspected in open label- studies so a risk factor is associated with it as it can influence microbe viability and effect on the host can be altered [113].

## 7. Fate of probiotic activity during major formulations

Probiotic formulations have been utilized as dietary supplements for several years. For a successful probiotic product, specific criteria must be met; optimization of the number of viable cells in Formulation is one of them. Liquid or semisolid formulations are the most common forms of probiotic products available in the market. The probiotic products carrying definite probiotic strain are developed in various formulations like fermented milk [116], sachets [117], chewing gum [118], and capsule*s* [119]. The fate of probiotic activity depends on several factors such as pH, storage temperature, evaporation, oxygen, humidity, hydrogen peroxide, etc.

The temperature has a significant impact on bacterial strain growth, as is well documented. Bacterial strains are inactivated by low temperatures and killed by high temperatures. Thus, the temperature has a significant role in the probiotic microbial activity, as has been described by many researchers. In conjunction with the increase in temperature, there is also an increase in activity levels of various strains. Too much heat, on the other hand, renders the bacteria’s enzyme activity inactive. As described by [120], several factors in probiotics are affected by temperature. They showed that the fermentation time needed to reach the required H+ ion concentration is proportional to the temperature. The higher the temperature, the lower the fermentation time required to achieve the necessary H+ ion concentration. Similarly, the titratable acidity of the probiotic is also inversely proportional to the temperature at which the fermentation is carried out [121].stated that the biomass concentration produced by the probiotic bacteria during fermentation is also temperature-dependent. They showed that the *Lactobacillus casei* species showed higher biomass concentration than other lactobacillus species, notably at 35°C and 37°C [122], showed the effect of temperature on the growth of bacteria and the production of the acid in the goat yogurt probiotic. Overall, the cumulative bacterial count grew significantly within 3 hours and slowed down at each temperature over the next 24 hours. The overall bacterial counts were 1.26, 1.36, 1.8, 1.85, and 1.65 (109 CFU/mL) at 37, 39, 41, 43, and 45°C, respectively. The acidity was seen to be moderate at 43°C. It was also seen that the probiotic (yogurt) had a strong smell if it was fermented at a lower temperature (which is 37°C) and had a good taste and state if fermented at a higher temperature. The development of beverage formulation as a probiotic carrier is a complicated process. Designing a product with probiotics is crucial for a strain to persist low and high pH. The most critical factor is that the probiotic must first survive the unfavorable effect of the gastric pH and then bile salts in the small intestine to ensure maximum health benefits. To survive, various techniques are used; microencapsulation is used to survive the stomach’s acidic pH [123]. Probiotic strains have different survival rates at different pH; for example, *Lactobacillus reuteri* DSM 17938 when cultured at pH 6.5, the survival rate of cells was highest while at pH 4.5, the survival rate was lowest [124] while in the case of *Lactobacillus rhamnosus* cells when grown at pH 5.0 found to have better survival rate than grown at pH 5.8 [125].

## 8. Conventional fermented fruits, vegetables and beverages of India as a source of probiotic/functional food ingredients

Conventional fermented foods and beverages are widely consumed and have been a source of dietary nutrition since the dawn of time. It may be made using reasonably rudimentary procedures and tools in the home or a cottage enterprise [126,127]. Fermentation is a cost-effective and ancient food preparation and storage [128]. Thus fermented dishes and beverages have long been popular in India. Fermented foods made from regional food crops and other biological resources are prevalent throughout the Indian subcontinent. However, the product’s composition and the underlying ingredient vary by area [129]. A vast spectrum of fermented vegetable products is produced in India’s eastern Himalayan areas for bioprocessing biodegradable vegetables for preservation and later ingestion. Sinki, a native fermented radish taproot pit fermented product of Sikkim Himalayas, is a distinctive variety of lactic acid fermentation found in the food biopreservation. Sinki fermentation is accomplished by a variety of LAB, including *Leuconostoc plantarum, Leuconostoc brevis, Leuconostoc casei*, and *Leuconostoc fallax* [130]. Another typical fermented food found in India is Khalpi, a fermented cucumber product popular among Sikkim’s Brahmin Nepalis [131]. The microorganisms isolated from Khalpi fermentation comprise *L. Plantarum, Leuconostoc fallax and L. Brevis* [132]. In 2018, *Singh et al*. demonstrated the molecular characterization of isolated bacterial strains from Manipur’s Utonga-kupsu, Hentak, and Ngari fish species. *The researchers found bacillus* and *Staphylococcus* species with significant antibacterial and probiotic capabilities in all three fermented fish products. Antimicrobial, antioxidant, and probiotic properties are all present in the isolated product HNS60. Another example of a conventional fermented food product is Soidon; a fermented bamboo shoot tip is a staple food of Manipur made by the Meitei women. The process for preparing it begins with collecting Bamboo shoots from the shoot apex of *Teinostachyum wightii*, a bamboo species. The bottom sections of the casing, as well as the exterior housings, are removed. The entire tips are buried in a clay pot of water. The prior batch’s soidon, or sour liquid, is reintroduced as a starter (1:1 dilution) and kept for fermentation for 3–7 days at room temperature. The leaves of *Garcinia pedunculata Roxb*. (heibung, a local name) are added during the fermentation stage to improve the flavor of soidon [133]. The fermented soidon consists of the LAB’s of *L. Brevis, Lactococcus lactis and Leuconostoc fallax* [132]. Besides these, India’s prominent fermented foods comprise Mesu, Ngari, Ziang-sang, Ipoh, Gnocchi, Kinema, Bekang, etc. These are rich in microbial strains like Bacillus sp., *Saccharomyces cerevisiae*, Candida sp. and Lactobacillus sp [134].

The use of nondairy probiotic fermentation is possible for sweet lime and sugarcane juice [135]. Different products have been used to make juice enriched with phytochemical herbs such as Ashwagandha (*Camellia sinensis*), Oat, Green Tea (*Camellia sinensis*), and whey. Sweet lime and cane juice serve as a functional probiotic drink for lactose intolerant individuals. Kanji, a functional non-milk-based probiotic drink of Indian origin made of carrots and beetroot, is one of the rich sources of β-carotene, tocopherol, and ascorbic acid. The fermentation is mediated by the lactic acid bacteria, i.e., *L. plantarum, L. casei* and *L. acidophilus*. Haria is a cultural beverage produced in eastern-central India by the fermentation of milk [64]. Handia, fermented cooked rice, is a wine; undistilled rice beverage consumed in India predominantly by tribals [136]. Other rice-based beverages like Chhang [137] and Chicha [138] are also produced in India. Palm wine is a traditional drink made with the sap of various palm trees consumed in India. Cauliflower, mustard, and radish using probiotic bacteria Pediococcus sp. and Lactobacillus sp. produce Gundruk (Probiotic drink) in India [132,139].

## 9. Future perspectives

Today, probiotics can be found in a wide variety of food items and drinks, and it is a rapidly expanding industry that has broadened its horizons attributed to the reason that health issues have become an ongoing concern, drawing the attention of the consumers to these functional foods [140]. The present world is besieged by the lethal COVID-19 outbreak triggered by a novel coronavirus that puts forth the importance and awareness of ‘Immunity-based supplements,’ which can boost the proposed functional food-based beverage technology. Due to such prevalent reasons, there has been more discovery and development in conventional and modern generation probiotic foods and beverages. In the not-too-distant future, there is a high probability of more probiotic-based food supplements and drinks. With this imminent emerging prospect of probiotics as potential food, the primary consideration to be taken into account is health and safety. For decades, the basic principle of using probiotics has been that they do more benefit than harm to human health. Still, the advent of specific welfare issues, especially with the growing number of probiotic strains, has triggered a safety examination. For the concern expressed about the strain type, it would be safe to say that the strain used in making probiotic beverages must meet specific requirements imposed by the probiotic beverage’s composition. The development of probiotics is usually undesirable in the case of non-fermented drinks. However, this can be quite the opposite in the case of fermented beverages, where these probiotics prove to be very beneficial. As a result, selecting the appropriate probiotic strain for a particular food application makes a significant contribution. Therefore, choosing a strain with strong technical properties would be critical [141]. However, when it comes to strain selection, the essential protection criteria include that it must be of human origin, which has been sequestered from the human gastrointestinal tract. Also, they must not be infective or have any virulent factor, must not facilitate any disease-related behaviors, and at last, they must not contain any antibiotic resistance gene which can be transferred. Aside from these health and safety concerns, a regulatory mechanism that aims to protect consumers from false, inaccurate, and deceptive statements and harmonize the trading market is also an essential requirement [142].

## 10. Conclusion

The industrial probiotic products indubitably depict the most appropriate and the simplest way for improving the daily intake of dairy and nondairy-based beverages. The global market has shown increased sales of probiotic beverages in the last few years, especially dairy-based products. For the production of probiotic beverages, there is a potential to use cereals, vegetables, and fruits as substrates. Even so, a lot of study and research is required in the field regarding juices. Each country has a different set of rules and guidelines for the approval of human and clinical trials. To be used for effective industrial production, it is necessary to conduct relevant tests and understand a specific probiotic strain’s property and metabolite production. Although the specification and policies are continuously improving with time, the rapidly growing market of probiotic-based beverages demands more rigorous attempts to assure the safety of consumers.
